# Novel diagnostic model for bone metastases in renal cell carcinoma patients based on bone scintigraphy analyzed by computer-aided diagnosis software and bone turnover markers

**DOI:** 10.1007/s10147-021-02107-3

**Published:** 2022-02-04

**Authors:** Takeshi Ujike, Motohide Uemura, Taigo Kato, Koji Hatano, Atsunari Kawashima, Akira Nagahara, Kazutoshi Fujita, Ryoichi Imamura, Norio Nonomura

**Affiliations:** Department of Urology, Osaka University Graduate School of Medicine, 2-2 Yamadaoka, Suita Osaka, 565-0871 Japan

**Keywords:** Computer-assisted diagnosis system, Bone scan, Renal cell carcinoma, Bone metastases, Bone turnover marker

## Abstract

**Background:**

Computer-assisted diagnosis (CAD) systems for bone scans have been introduced as clinical quality assurance tools, but few studies have reported on its utility for renal cell carcinoma (RCC) patients. The aim of this study was to assess the diagnostic validity of the CAD system for bone scans and to construct a novel diagnostic system for bone metastases in RCC patients.

**Methods:**

We evaluated bone scan images of 300 RCC patients. Artificial neural network (ANN) values, which represent the probability of abnormality, were calculated by BONENAVI, the CAD software for bone scans. By analyzing ANN values, we assessed the diagnostic validity of BONENAVI. Next, we selected 108 patients who underwent measurements of bone turnover markers and assessed the combined diagnostic validity of BONENAVI and bone turnover markers.

**Results:**

Forty-three out of 300 RCC patients had bone metastases. The AUC of ANN values was 0.764 and the optimum sensitivity and specificity were 83.7 and 62.7%. By logistic analysis of 108 cases, we found that ICTP, a bone resorption marker, could be a diagnostic marker. The AUC of ICTP was 0.776 and the optimum sensitivity and specificity were 57.1 and 86.8%. Subsequently, we developed a novel diagnostic model based on ANN values and ICTP. Using this model, the AUC was 0.849 and the optimum sensitivity and specificity were 76.2 and 80.7%.

**Conclusion:**

By combining the high sensitivity provided by BONENAVI and the high specificity provided by ICTP, we constructed a novel, high-accuracy diagnostic model for bone metastases in RCC patients.

## Introduction

Bone metastases are found in approximately 30% of advanced renal cell carcinoma (RCC) patients, representing the second- most common site of distant metastasis (following lung) [1, 2]. Because such lesions profoundly impact quality of life and survival [3], early diagnosis of bone metastases is clinically important. However, there are no firm guidelines for diagnosis of bone metastasis in RCC patients.

Bone scans are highly sensitive methods for detecting bone metastasis, and are also an effective diagnostic tool for whole-body examinations. Therefore, bone scans are widely used for identifying metastases from various cancers, but it has been reported that they have limitations in detecting osteolytic-type bone metastases, which is the predominant type in RCC [4]. Thus, clinical guidelines for RCC do not recommend bone scans routinely [5, 6].

Recently, a computer-assisted diagnosis (CAD) system for bone scans was introduced as a clinical quality assurance tool. There are many reports that the CAD system is useful for both diagnosis and prognostic prediction with respect to prostate cancer exhibiting osteogenic bone metastasis [7–9]. However, there are few reports on the usefulness of RCC exhibiting osteolytic bone metastasis.

In the diagnosis of bone metastasis of various cancers, many studies on the usefulness of bone turnover markers as a tool for quantitative diagnosis have been made, although this also remains controversial [10–12].

The aim of this study was to assess the diagnostic validity of the CAD system for bone scans and bone turnover markers, and ultimately to develop a novel diagnostic model for bone metastases in RCC patients.

## Materials and methods

### Patients characteristics

We retrospectively selected 300 RCC patients who underwent bone scintigraphy in Osaka University Hospital from January 2008 through May 2016. Every patient was classified into one of two groups—those with and those without bone metastases—based on information from multiple modalities including X-ray, computed tomography (CT), magnetic resonance imaging (MRI), positron emission computed tomography (PET), biopsy result, serial bone scans and other follow-up studies. Using this cohort, we first evaluated the ability of the CAD system to diagnose bone metastases in RCC patients.

Subsequently, we analyzed bone turnover marker data that were available from 104 of the 300 patients. Using this cohort, we examined whether the CAD system and bone turnover markers are useful for diagnosis of bone metastases. Finally, we attempted to construct a novel diagnostic system using factors determined to be informative from logistic regression analysis results.

This study was approved by the Osaka University Hospital institutional review board (The IRB approval number is 12454).

### Bone scans

Patients were injected intravenously with 740 MBq 99 mTc MDP. At 3 h after injection, whole-body bone scans were obtained in the anterior view and the posterior view with a gamma camera equipped with low-energy high-resolution parallel hole collimators.

### CAD system

Raw imaging data sets were analyzed by software package BONENAVI version 2 based entirely on a database of individuals in Japan. This CAD system calculates two imaging metrics: one is an artificial neural network (ANN), and the other is bone scan index (BSI). ANN values represented the probability of abnormality. Input factors for ANN calculations were size, shape, intensity, and localization of RI accumulation. An ANN value for each hot spot was calculated by BONENAVI, and this value determined whether a spot was classified as a bone metastasis or not. BONENAVI presented an output between 0 and 1 for each patient as ANN values. ANN values in the range of 0–0.50 were categorized as benign, while those ranging from 0.51 to 1.00 were categorized as bone metastases. BSI was calculated as the percentage of the sum of all spots classified as bone metastasis in a patient’s body. In this study, BSI was not used because the aim of this study was to examine the diagnostic ability of BONENAVI.

### Bone turnover markers

We selected six bone turnover markers: (i) Bone-formation markers, including alkaline phosphatase (ALP) and bone-specific alkaline phosphatase (BAP) and (ii) Bone resorption and osteoclastogenesis markers, including Pyridinoline cross-linked carboxyterminal telopeptide of type I collagen (ICTP), urinary *N*-telopeptide (uNTx), serum *N*-telopeptide (sNTx), and Tartrate-resistant acid phosphatase 5b (TRACP-5b). In addition to these bone turnover markers, we examined eight factors including serum calcium (Ca) and lactate dehydrogenase (LDH).

### Statistical analysis

Results were expressed as the median (range) for continuous variables. Univariate analysis was performed by the Mann–Whitney *U* test or the chi-square test. Univariate and multivariate logistic regression analyses were performed to determine the correlation between presence of bone metastases　and clinical factors (ANN values and bone turnover markers). The predicted probability of bone metastases was estimated as *P* = 1/ (1 + e^−*x*^). Logistic regression yields a score (*X*), where *X* is β_0_ + β_1_X_1_ + β_2_X_2_ + β_3_X_3_…, which is a linear combination of the predictors (X_1_, X_2_, X_3_…) in the model. The model coefficients (β_0_, β_1_, β_2_…) were chosen to optimize the ability to predict presence of bone metastases. A nomogram predicting the probability of presence of bone metastases was constructed based on this formula. This new diagnostic model was evaluated for diagnostic ability using the receiver-operator characteristics (ROC) curve analysis. Statistical significance was considered as *p* < 0.05. All data analyses were performed with JMP ver.14 (SAS Institute Inc.,Cary, NC, USA).

## Results

**Fig. 1:**
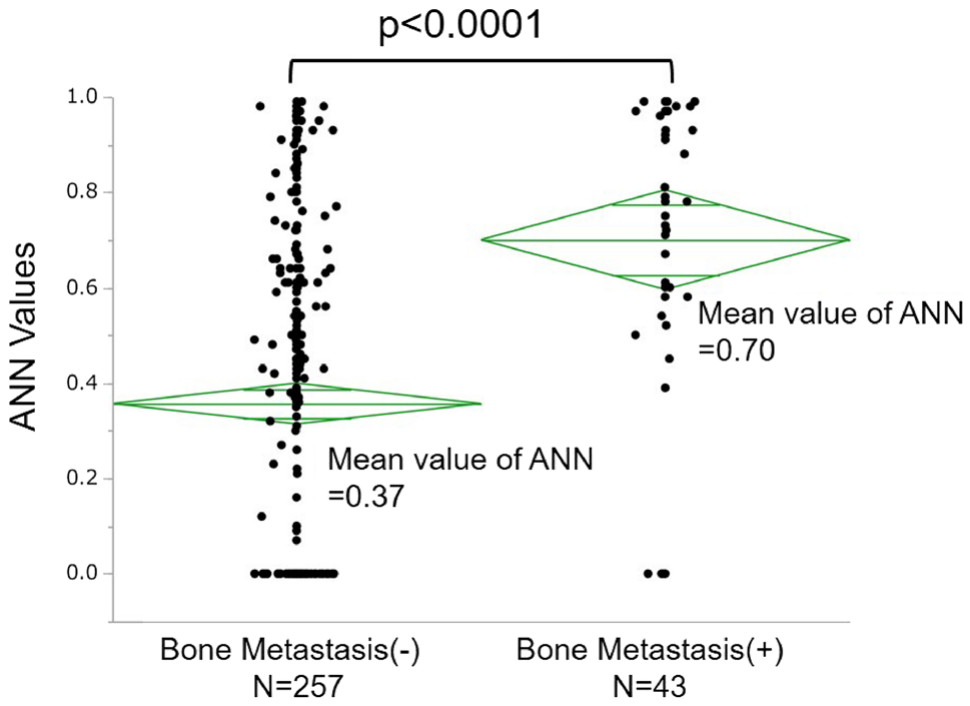
The plots of the raw data correspond to the true positive of ANNs for RCC patients with bone metastases or without bone metastases

**Fig. 2:**
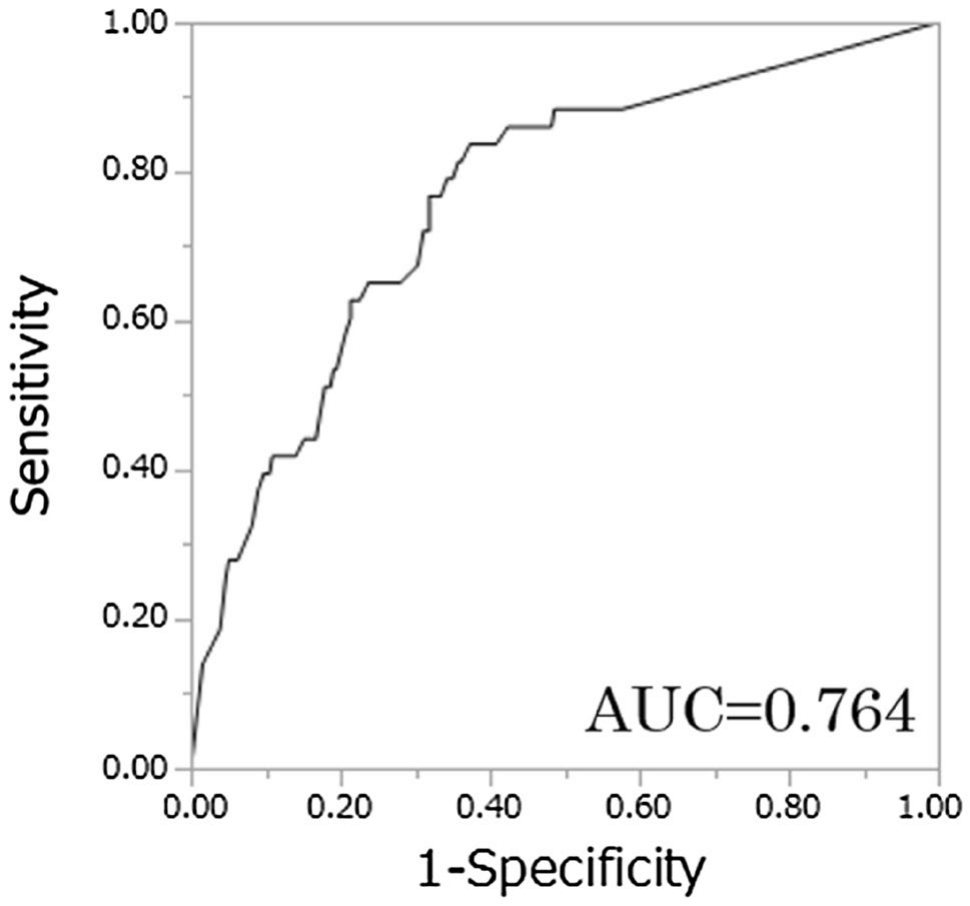
The receiver operating characteristic (ROC) curves describing the diagnostic performance of BONENAVI to identify bone metastases

Patient characteristics are summarized in Table 1. Among 300 patients, 43 patients (14.3%) had metastatic bone tumors. The mean ANN values of the patients with bone metastases were significantly higher than those without bone metastases (0.70, 0.37, respectively. *P* < 0.0001) (Fig. 1). The AUCs for the diagnosis of a patient with bone metastasis by BONENAVI were 0.764 and the optimum sensitivity and specificity were 83.7 and 62.7%, respectively (Fig. 2).

**Table 1:** Patients characteristics of all cases

Variables	Metastatic bone tumor	
	Negative	Positive
Number	257	43
Median age, year (range)	64 (27–94)	68 (27–86)
Gender male/female	181/76	36/7
Tumor histology, *n* (%)		
Clear cell	170 (66.0)	23 (53.4)
Papillary	2 (0.8)	2 (4.7)
Chromophobe	4 (1.6)	2 (4.7)
Spindle	3 (1.2)	0 (0)
Unclassified	3 (1.2)	0 (0)
Unknown	71 (29.2)	16 (37.2)

**Fig. 3:**
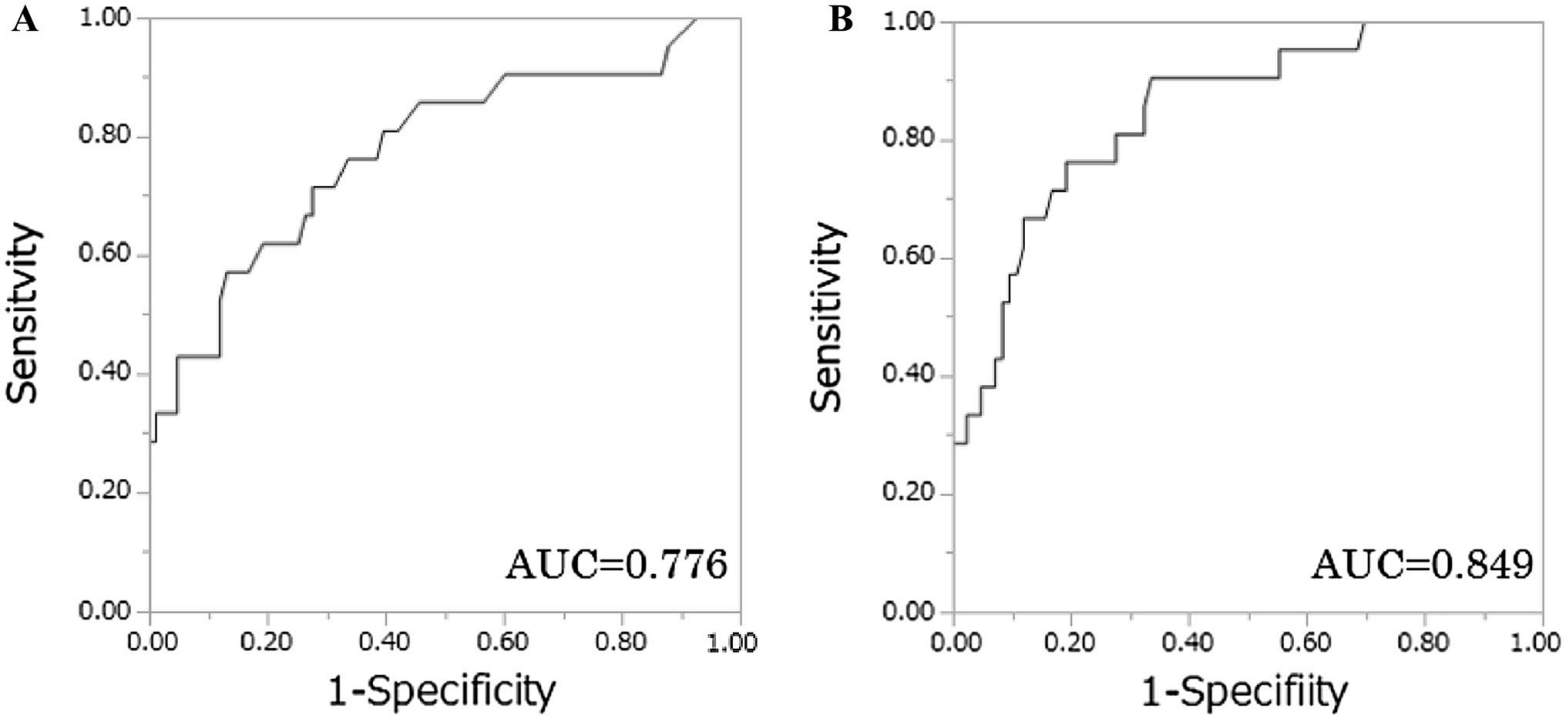
The receiver operating characteristic (ROC) curves describing the diagnostic performance of ICTP **A** and novel diagnostic model **B** to identify bone metastases

Subsequently, we assessed the ability of bone turnover markers to predict bone metastases using clinical data from 104 patients. Patient characteristics are summarized in Table 2. Among 104 patients, 21 patients (20.2%) had metastatic bone tumors. In univariate analysis, ICTP of the groups with bone metastases were significantly higher compared with those without bone metastases. The other factors (BAP, uNTx, sNTX, TRACP5b, ALP, Ca, LDH) were not significantly different between two groups. Multivariate logistic regression analysis revealed that ICTP was an independent predictor for presence of bone metastases (*p* < 0.0001), whereas sNTX were not significant (Table 3). The AUCs for the diagnosis of a patient with bone metastasis by ICTP were 0.776 and the optimum sensitivity and specificity were 57.1 and 86.8%, respectively (Fig. 3A).

**Table 2:** Patient characteristics of 104 patients who were measuring bone turnover markers

Parameter		Metastatic bone tumor		*P* value
		Negative	Positive	
Number		83	21	
Median age, year (range)		66 (40–94)	71 (45–86)	0.373
Gender	male/female	53/30	18/3	0.055
Stage	I/II/III/IV/unknown	42/4/21/14/2	0/1/4/16/0	< 0.0001
Tumor histology clear cell/papillary/chromophobe/unclassified/unknown		72/0/2/3/6	11/3/1/3/3	0.0008
Median values of bone turnover markers, (range)				
ICTP	(ng/mL)	4.3 (2.2–15.1)	7.2 (2.8–38.1)	< 0.0001
BAP	(μg/L)	12.3 (3.5–51.8)	13.5 (7.5–34.4)	0.252
uNTx	(nmolBCE/mmolCr)	30.0 (5.7–129.6)	30.6 (6.9–96.1)	0.536
sNTx	(nmolBCE/mmolCr)	15.6 (8.7–30.0)	16.2 (8.6–35.3)	0.366
TRACP-5b	(mIU/dL)	330 (109–898)	353 (87–977)	0.606
ALP	(IU/L)	225 (70–2406)	262 (162–1010)	0.015
Ca	(mg/dL)	9.2 (8.2–14.6)	9.5 (8.4–12.0)	0.142
LDH	(IU/L)	193 (107–532)	201 (137–542)	0.175

*ICTP* Pyridinoline cross-linked carboxyterminal telopeptide of type I collagen, *BAP*  bone-specific alkaline phosphatase, *uNTx *urinary *N*-telopeptide, *sNTx * serum, *N*-telopeptide, *TRACP-5b *Tartrate-resistant acid phosphatase 5b, *ALP *alkaline phosphatase, *Ca *serum calcium, *LDH *lactate dehydrogenase, *P* value was calculated by Mann–Whitney *U* test or χ-square test

**Table 3:** Logistic analysis of variables associated with detection of bone metastases in 104 cases

Parameter	Univariate			Multivariate		
	OR	95% CI	*P* value	OR	95% CI	*P* value
ICTP	1.29	1.14–1.50	< 0.0001	1.31	1.14–1.56	< 0.0001
BAP	1.03	0.96–1.10	0.389			
uNTx	1.01	0.99–1.04	0.328			
sNTx	1.07	0.99–1.17	0.093	0.95	0.84–1.07	0.410
TRACP-5b	1.00	0.99–1.00	0.324			
ALP	1.00	0.99–1.00	0.589			
Ca	1.27	0.70–2.22	0.399			
LDH	1.00	0.99–1.00	0.267			

*OR * odds ratio, *CI* confidence interval, *ICTP*  Pyridinoline cross-linked carboxyterminal telopeptide of type I collagen, *BAP*  bone-specific alkaline phosphatase, *uNTx*  urinary, *N*-telopeptide, *sNTx* serum, *N*-telopeptide, *TRACP-5b* Tartrate-resistant acid phosphatase 5b, *ALP *alkaline phosphatase, *Ca *serum calcium, *LDH* lactate dehydrogenase *P* value was calculated by Likelihood-ratio test

Finally, by combining the high sensitivity provided by ANN values and the high specificity provided by ICTP levels, we attempted to construct a novel, high-accuracy diagnostic model for bone metastases in RCC patients. From the result of multivariate logistic regression analysis using ANN values and ICTP levels, we created a novel diagnostic model for the probability of presence of bone metastases as represented by the following formula: *P* = 1/ (1 + e^−*x*^).

*X* = 3.12-0.890 × ANNscore-0.230 × ICTP(ICTP: continuous variables, ANN score: ANN > 0.5 assigns ANN score of 1, ANN≦0.5 assigns ANN score of 0). Using this diagnostic model (ICTP-ANN model), the AUC for the probability of presence of bone metastases was 0.849. The optimum sensitivity and specificity were 76.2 and 80.7%, respectively (Fig. 3B).

## Discussion

Identification of bone metastases is essential for making informed treatment decisions for any malignant tumors. Despite the increasing number of reports concerning various imaging modalities in the diagnosis for bone metastases, the accuracy of each modality, specifically X-ray, CT, MRI, bone scans and PET-CT, still remains unknown and no consensus has been reached. Despite the advent of various imaging modalities, whole-body bone scans remained the standard method for surveying the presence and extent of bone metastasis.

Since bone scans are sensitive in detecting changes in osseous metabolism, it is difficult to distinguish malignant tumors from inflammatory changes and other biological phenomena. As a result, bone scans can be prone to false positives. In addition, analysis of bone scans can be highly subjective, making it difficult to obtain diagnoses that are reproducible across multiple clinicians.

To eliminate the need for operator discretion, CAD software, EXINIbone (EXINI Diagnostics AB, Lund, Sweden), for the automated detection and quantification of imaging data, was developed in 2006 [13]. BONENAVI (Fujifilm RI Pharma Co. Ltd., Tokyo), the CAD system adopted in this study, utilizes the same system as EXINIbone. The initial CAD system, EXINIbone, was constructed using a Swedish database. BONENAVI ver.1, was constructed by adopting 904 Japanese patients from a single institution’s database [14–16]. BONENAVI ver2, the latest CAD system used in this study, was trained using 1532 Japanese patients from a database comprising nine institutions [17–19]. Diagnostic accuracy using BONENAVI ver2. was superior to those using EXINIbone and BONENAVI ver.1 [17].

BONENAVI generates two imaging markers: an artificial neural network (ANN) value, and a bone scan index (BSI). The ANN value shows the probability of having skeletal metastasis, and the BSI value shows the bone metastatic tumor burden. Many reports have claimed prognosis could be predicted in prostate cancer patients using these two values [9, 20–23].

**Fig. 4:**
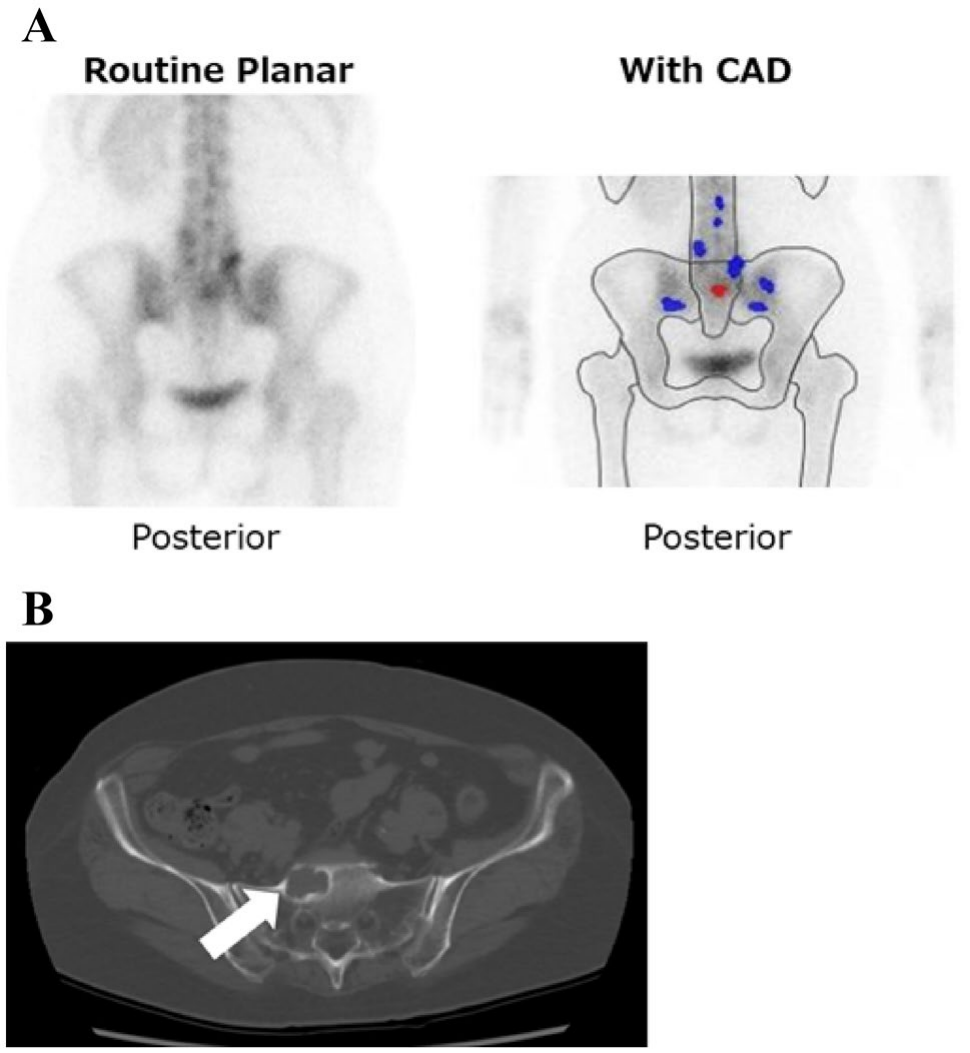
A 74-year-old female pointed out sacral metastasis nine months after radical nephrectomy. Although RI accumulation of sacral in the routine planar is not clear, it could be clearly judged with BONENAVI. Computed tomography could point out osteolytic changes (white arrow)

On the other hand, there are no reports that examined the usefulness of BONENAVI for diagnosis of bone metastasis in RCC patients. Bone metastasis in RCC patients often exhibits osteolytic properties, and it is speculated that it is difficult to detect malignancy by bone scan only. However, we experienced a case where BONENAVI was very useful for diagnosis of bone metastasis (Fig. 4). In this case, BONENAVI clearly confirmed the RI accumulation that was difficult to confirm in the routine planar. Finally, a definitive diagnosis was made by histological examination by CT-guided needle biopsy. We hypothesized that BONENAVI could also be useful in diagnosing bone metastases in RCC patients.

In this study, BONENAVI alone had high sensitivity (83.7%) on diagnosis for bone metastases, and a certain degree of diagnosis ability was observed. However, the specificity was as low as 62.7%, which was not a satisfactory range. Therefore, to supplement this weak point, we decided to combine the BONENAVI metrics with bone turnover marker measurements, which have been well studied.

For many years, studies have been conducted on various cancers regarding the usefulness of measuring bone turnover markers at the time of diagnosis of bone metastases, but no conclusion has yet been reached [10–12]. On the diagnosis of bone metastases in RCC patients, there are few reports on whether bone turnover markers are useful. Jung et al. assessed the diagnostic accuracy of bone turnover markers in the serum of 72 RCC patients [24]. They examined whether ALP, NTx, TRACP5b, OPG (osteoprotegerin), and RANKL (ligand of the receptor activator of nuclear factor-κB) were useful as diagnostic markers for bone metastases. They conclude that bone turnover markers are hardly useful to diagnose bone metastases in RCC patients. However, their study did not assess ICTP.

In this study, we found that ICTP is the most useful among the bone turnover markers we examined. ICTP is a degradation product of type I collagen and is associated with the bone resorption process. It is scientifically consistent that serum ICTP is elevated in RCC patients with bone metastases characterized by osteolytic changes. In the context of lung cancer, reports have claimed that ICTP was higher in patients with bone metastases than those without bone metastases [25]. However, a limited number of cases have been examined, and no conclusion has been reached regarding the usefulness of ICTP.

By combining the high sensitivity provided by ANN values and the high specificity provided by ICTP levels, we were able to construct a novel diagnostic system with high AUC values (= 0.849). However, several limitations should be taken into consideration. First, this study consisted of only Japanese patients, and these results may not be applicable to other races. Second, the number of patients who had bone metastasis in this study was relatively small. Third, our study was a retrospective and single-center study. However, we strongly believe that these interesting results can help clinicians treating RCC patients with suspected bone metastases. Of course, to establish the novel diagnostic system of this study, we believe that further large-scale studies are needed.

## Conclusion

We confirmed that the diagnostic ability of BONENAVI for bone metastases in RCC patients is highly sensitive. After confirming this result, we constructed a novel, high-accuracy diagnostic tool for bone metastases in RCC patients by combining the high sensitivity provided by BONENAVI and the high specificity provided by ICTP levels.
